# Evaluation of dose coverage to target volume and normal tissue sparing in the adjuvant radiotherapy of gastric cancers: 3D-CRT compared with dynamic IMRT

**DOI:** 10.2349/biij.6.3.e29

**Published:** 2010-07-01

**Authors:** KK Murthy, KA Shukeili, SS Kumar, CA Davis, RR Chandran, S Namrata

**Affiliations:** 1 Department of Radiotherapy, Krishna Institute of Medical Sciences, Hyderabad, India; 2 Department of Radiotherapy, National Oncology Centre, The Royal Hospital, Muscat, Sultanate of Oman

**Keywords:** Conformal radiotherapy, intensity modulated radiotherapy, organs at risk, planned target volume, tolerance doses

## Abstract

**Purpose:**

To assess the potential advantage of intensity-modulated radiotherapy (IMRT) over 3D-conformal radiotherapy (3D-CRT) planning in postoperative adjuvant radiotherapy for patients with gastric carcinoma.

**Methods and materials:**

In a retrospective study, for plan comparison, dose distribution was recalculated in 15 patients treated with 3D-CRT on the contoured structures of same CT images using an IMRT technique. 3D-conformal plans with three fields and four-fields were compared with seven-field dynamic IMRT plans. The different plans were compared by analyzing the dose coverage of planning target volume using TV_95_, D_mean_, uniformity index, conformity index and homogeneity index parameters. To assess critical organ sparing, D_mean_, D_max_, dose to one-third and two-third volumes of the OARs and percentage of volumes receiving more than their tolerance doses were compared.

**Results:**

The average dose coverage values of PTV with 3F-CRT and 4F-CRT plans were comparable, where as IMRT plans achieved better target coverage(*p*<0.001) with higher conformity index value of 0.81±0.07 compared to both the 3D-CRT plans. The doses to the liver and bowel reduced significantly (*p*<0.001) with IMRT plans compared to other 3D-CRT plans. For all OARs the percentage of volumes receiving more than their tolerance doses were reduced with the IMRT plans.

**Conclusion:**

This study showed that a better target coverage and significant dose reduction to OARs could be achieved with the IMRT plans. The IMRT can be preferred with caution for organ motion. The authors are currently studying organ motion in the upper abdomen to use IMRT for patient treatment.

## INTRODUCTION

Gastric cancer is the second most common cancer worldwide, with a frequency that varies greatly across different geographic locations [1]. Despite the decreasing worldwide incidence, gastric cancer accounts for 3% to 10% of all cancer related deaths [2]. In spite of technical advances in surgery and adjuvant therapy, the mortality associated with gastric cancer is prevailing. Patients with localized node negative gastric cancer have 5-year survival rates that approach 75% when treated with surgery alone [3]. This is in contrast to patients with lymph node involvement, in whom survival rates range from 10% to 30% [4, 5, 6]. Preliminary studies of adjuvant chemo-radiotherapy showed promising results in patients resected with curative intent [7, 8].

The target delineation as well as the treatment technique of radiation dose delivery to the post operative stomach remains complex. This is due to the large planned target volume (PTV) and surrounding mobile parts of bowel and other critical organs, such as liver, kidneys and spinal cord. Recent data indicate that post-operative chemo-radiotherapy improves clinical outcome by improving the relapse-free survival in gastric cancer and that acute toxicity is acceptable [9]. Data on late side effects, however, are scarce. Renal function impairment represents one of the most serious late complications following abdominal radiotherapy.

The CT images with 3-dimensional (3D) planning software help in displaying the 3D-dose distribution at different levels in the PTV. In conventional 3D-CRT for stomach cancer, very commonly three-field technique (3F-CRT) or four-field technique (4F-CRT) is used. All the fields are shaped with MLC and either physical or dynamic wedges are used to achieve optimal three-dimensional dose distribution with minimal degree of dose inhomogeneity through forward treatment planning. Early and late radiation induced complications are directly related to the total dose delivered, fractionation scheme, radiation treatment technique and patient anatomy. Several institutions have reported the use of different techniques to improve the dose distribution within the PTV [10-13].

Despite all efforts in the conventional method of planning, the surrounding normal tissue of PTV and other critical organs at risk still receives considerable doses in the final plan. In Intensity Modulated Radiotherapy (IMRT) it is possible to overcome this problem by achieving desired dose distribution with its ability to provide sharp dose gradients at the junction of target volume and the adjacent critical organs. In order to explore the advantages of IMRT treatment and extend it to the gastric cancer patients, a retrospective study was carried out by the first author (KMM) at his previous institution on 15 patients of gastric cancer treated with 3D-CRT. In this article the authors describe the planning methods used and show the comparison of results for 4F-CRT and IMRT plans of a representative patient. They also furnish DVH comparison results of conventional 3F-CRT, 4F-CRT and dynamic IMRT for all 15 cases.

## MATERIALS AND METHODS

3D Radiation Treatment Planning System (RTPS) Eclipse (version 6.5, Varian Ag, USA) with Helios inverse planning software was used for treatment planning. High energy Linear Accelerator Clinac 2300 CD (Varian Ag, USA) having 120 leaf millennium MLC was used for the delivery of treatments. Fifteen patients planned and treated with three/four field 3DCRT were taken up for a retrospective study by re-planning with dynamic IMRT technique and comparing the dose distributions in PTV and organs at risk. For all the cases radiation was given in 25 fractions of 1.8 Gy to a total dose of 45 Gy in 5 weeks. CT images of 5mm thickness at different transverse sections away from the mid plane were taken to create a 3D image. Initially the 3D forward planning of 3F-CRT with three fields (AP and two laterals) was done for 15 patients in conventional way. Then 4F-CRT planning with four fields (AP, two laterals and PA) was done for each patient. In both cases, appropriate wedges were used to obtain uniform dose distribution in the target volume. The beam energies (6 and 15MV), beam weightings and MLC leaf positions were optimized by forward planning to reduce the doses to the critical organs and achieve a better homogeneous dose distribution in the PTV.

Following the 3DCRT plans, dynamic IMRT plans were created on the same CT images with structures. Seven fields of 6MV energy with equal separation of gantry angles were used. A constraints template was created and applied to all the 15 patient plans. Wherever required and achievable, the constraints were changed to obtain possible minimum doses to critical organs without compromising the PTV coverage of at least 95% dose to 95% of PTV volume.

**Comparison parameters:** To asses the target coverage and normal tissue sparing the following parameters were used.

1. A uniformity index was used and defined as:
  (1) $$UI=\frac{D_{5}}{D_{95}}$$
  Where D_5_ and D_95_ are the minimum doses delivered to 5% and 95%, respectively of the PTV as previously described by Wang et al. [14] and Kesheng et al. [15]. In addition, to assess target coverage, the mean and maximum doses to PTV, percentage of target volume receiving at least 95% of the prescribed dose TV_95_(%) and the dose to 1% of target volume D_1_(%) were calculated.
2. Radiation dose homogeneity index (HI), which was defined by Nutting et al. [16] and Pezner et al. [17] as the difference in PTV dose between D_1_ and D_99_ divided by the prescription dose was calculated. Smaller HI corresponds to more homogeneous dose distribution in PTV.
3. The conformity index, CI was calculated by using the following formula [18].
  (2) $$CI=\left(\frac{TV_{95}}{TV}\right)\left(\frac{TV_{95}}{V_{95}}\right)$$
  where, TV_95_ is the volume of target covered by the 95% isodose line, TV is the total target volume and V_95_ is the volume of tissue covered by the 95% isodose line. The value of CI varies between 0 to 1 and a value close to 1 gives better conformity of dose to the PTV.
4. The sparing of the organs at risk was evaluated by comparing their maximum and mean doses. D_2/3_ and D_1/3,_ defined as the dose received by 2/3 and 1/3 volumes of the organ respectively, were also analysed for tolerance limits [19, 20].
5. The percentage of volumes of organs at risk (OARs) receiving a dose more than their corresponding tolerance limit (V_>TL_) were compared.

The values of the above parameters of 15 cases planned by conventional forward planning with 3F-CRT, 4F-CRT and the dynamic IMRT technique were compared with the help of their dose volume histograms. Statistical analysis was performed with the two-tailed paired t-test. A p-value of p<0.05 was considered statistically significant.

## RESULTS

**Figure 1:**
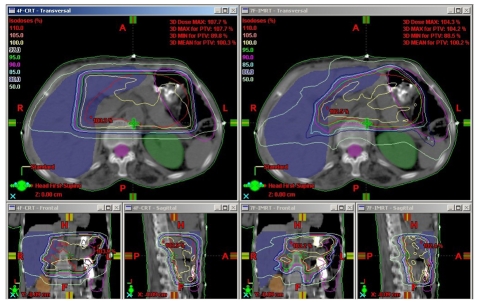
Isodose curves on an axial slice at isocenter plane of a representative patient for 4F-CRT and IMRT. The lower part of the figure shows the isodose distribution on the coronal and sagittal plane of the corresponding slice.

**Figure 2:**
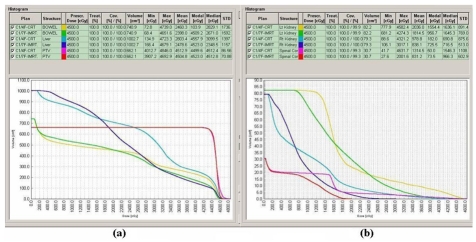
Comparison of DVH curves of 4F-CRT and IMRT plans of a representative patient for (a) PTV, Liver and Bowel, (b) Left kidney, Right Kidney and Spinal cord.

All three planning techniques produced acceptable dose distributions to the planned target volume. All the dose coverage parameters of PTV for the 3F-CRT and 4F-CRT plans showed similar and comparable values without significant differences. The isodose distributions obtained on an axial slice at the isocenter plane of a representative patient for 4F-CRT and IMRT are shown in Figure 1. The plan comparison DVH curves for PTV and OARs of the same patient for 4F-CRT and IMRT are shown in Figure 2. The analysed data of fifteen patients with the mean doses to the PTV and comparison of dose coverage with 3D-CRT and IMRT treatment plans is shown in Table 1. The results indicate that there was a statistically significant and considerable difference in the dose coverage of PTV with IMRT (*p*<0.001) compared to both 3D-CRT plans. The average lower values of SD, UI, HI and higher values of CI for IMRT plans compared to the 3F-CRT and 4F-CRT plans confirms the advantage of IMRT plans over both 3D-CRT plans.

**Table 1:** Comparison of the average dose parameters of 15 patients for the PTV among the three planning techniques.

**PTV**	**3F-CRT**	**4F-CRT**	**IMRT**	**P-Value**		
				**3F-CRT vs IMRT**	**4F-CRT vs IMRT**	**3F-CRT vs 4F-CRT**
Dmean (%)	101.80 ± 1.31	101.85 ± 1.02	100.44 ± 0.45	<0.001	<0.001	0.914
Dmax (%)	108.96 ± 1.45	108.81 ± 1.03	106.09 ± 1.28	<0.001	<0.001	0.751
D_1%_ (%)	107.00 ± 1.48	106.85 ± 1.11	103.89 ± 0.77	<0.001	<0.001	0.699
TV_95%_ (%)	98.79 ± 1.31	98.95 ± 1.07	97.94 ± 1.29	0.08	0.026	0.697
SD (%)	2.43 ± 0.50	2.37 ± 0.54	1.91 ± 0.37	0.003	0.011	0.753
UI	1.08 ± 0.02	1.08 ± 0.02	1.06 ± 0.01	<0.001	0.005	0.388
CI	0.62 ± 0.04	0.61 ± 0.04	0.81 ± 0.07	0.039	<0.001	0.839
HI	0.12 ± 0.02	0.11 ± 0.02	0.10 ± 0.02	<0.001	0.108	0.692

Dmean, mean dose; Dmax, maximum dose; D_1%_, dose to 1% of target volume; TV_95%_, dose to 95% of target volume; UI, uniformity index; CI, conformity index; HI, homogeneity index; SD, standard deviation of dose in the PTV.

The dose coverage of OARs with 3F-CRT, 4F-CRT and IMRT plans along with the p-values are shown in Table 2. The average mean dose values of liver were less in 4F-CRT plans compared with that in 3F-CRT plans. There was however, a significant (*p*<0.001) dose reduction in liver with IMRT plans compared to both 3D-CRT plans.

**Table 2:** Comparison of average dose distribution in the Organs at risk (OARs) for a prescribed dose of 45Gy for 15 patients.

**OAR**	**Tolerance Dose limit TD 5/5 (Gy)**	**Mean Dose ± SD (%)**			**P-Value**		
		**3F-CRT**	**4F-CRT**	**IMRT**	**3F-CRT vs IMRT**	**4F-CRT vs IMRT**	**3F-CRT vs 4F-CRT**
**Liver**							
D_max_		48.1 ± 1.0	47.7 ± 0.8	47.0 ± 1.0	0.008	0.038	0.318
D_mean_		28.0 ± 3.9	26.9 ± 3.9	23.7 ± 3.5	<0.004	0.025	0.428
D_2/3_	30	25.4 ± 6.0	23.4 ± 6.1	17.6 ± 4.8	<0.001	0.007	0.368
D_1/3_	35	36.9 ± 3.4	34.1 ± 3.7	28.1 ± 3.5	<0.001	<0.001	0.047
V_>30Gy_	50	26.8 ± 5.3	23.0 ± 5.3	13.1 ± 3.5	<0.001	<0.001	0.055
**Right Kidney**							
D_max_		44.9 ± 2.4	44.8 ± 4.0	35.8 ± 3.5	<0.001	<0.001	0.92
D_mean_		12.0 ± 2.6	12.9 ± 2.5	11.6 ± 1.5	0.594	0.1	0.353
D_2/3_	23	6.3 ± 3.5	6.1 ± 3.7	7.3 ± 1.8	0.317	0.246	0.877
D_1/3_	30	13.6 ± 1.8	16.4 ± 2.9	12.3 ± 1.2	0.021	<0.001	0.004
V_>23Gy_	36	5.7 ± 4.0	6.3 ± 4.0	2.4 ± 1.3	0.005	0.001	0.687
**Left Kidney**							
D_max_		44.2 ± 2.1	44.8 ± 2.2	37.7 ± 4.5	<0.001	<0.001	0.463
D_mean_		13.7 ± 3.1	15.0 ± 2.6	14.5 ± 2.1	0.388	0.61	0.227
D_2/3_	23	10.1 ± 2.8	11.5 ± 3.8	11.1 ± 2.7	0.333	0.775	0.284
D_1/3_	30	14.3 ± 2.2	17.3 ± 2.1	17.3 ± 2.5	0.002	0.989	<0.001
V_>23Gy_	36	5.5 ± 3.3	6.7 ± 3.8	7.3 ± 2.1	0.27	0.739	0.368
**Spinal cord**							
D_max_		22.8 ± 9.9	25.1 ± 7.5	25.2 ± 2.2	0.369	0.974	0.474
D_mean_		9.8 ± 1.9	11.6 ± 2.2	11.0 ± 1.9	0.086	0.395	0.018
D_2/3_	45	5.8 ± 4.4	6.3 ± 4.9	4.6 ± 4.1	0.448	0.33	0.796
D_1/3_	50	13.1 ± 1.7	16.4 ± 1.8	16.0 ± 2.1	<0.001	0.358	<0.001
V_>45Gy_	50	0.0 ± 0.0	0.0 ± 0.0	0.0 ± 0.0	0.115	0.153	0.944
**Bowel**							
D_max_		49.0 ± 0.8	48.7 ± 0.7	47.1 ± 0.7	<0.001	<0.001	0.297
D_mean_		27.5 ± 4.4	27.0 ± 4.3	24.6 ± 3.6	0.058	0.099	0.785
D_2/3_	40	22.1 ± 6.4	22.0 ± 5.5	17.9 ± 2.9	0.029	0.015	1
D_1/3_	45	36.6 ± 4.9	35.8 ± 5.7	31.5 ± 2.4	0.001	0.011	0.684
V_>40Gy_	50	12.9 ± 4.6	13.2 ± 4.7	9.8 ± 3.3	0.046	0.03	0.857

The average mean values and other doses of both the kidneys and the spine with the 3F-CRT plans were lower than that with the 4F-CRT plans. The reduction of liver dose in 4F-CRT and reduction of doses in kidneys and spine with 3F-CRT plans were attributed to the addition of the PA field in the 4F-CRT plans. The IMRT plans were able to achieve lower values of doses in the right kidney compared to that of both 3D-CRT plans, whereas the dose values in the left kidney were comparable. Similarly the dose values in the spinal cord with IMRT plans were lower than that with the 4F-CRT plans and slightly higher than that with the 3F-CRT plans.

In the case of bowel, all the mean dose values were similar and comparable in both 3D-CRT plans and they were significantly reduced in IMRT (*p*<0.001) plans. In all OARs the percentage of volumes receiving more than their tolerance doses were reduced significantly (*p*<0.001) with IMRT plans compared with both 3D-CRT plans.

## DISCUSSION

The toxicity associated with adjuvant radiotherapy using conventional 3DCRT techniques is significant. This is because of the standard target prescribed dose of 45 Gy well exceeds the tolerance of surrounding critical organs, namely kidneys and liver. Thus one has to compromise either in prescription to treat at tolerance doses of normal tissues rather than to the specific tumoricidal dose or in tailoring of the conventional treatment volumes. In both cases, local control and survival may be compromised. As IMRT delivers more conformal dose to the target by sparing surrounding critical structures, it allows complete target coverage to full dose and improves locoregional control and reduces toxicity. A number of studies have demonstrated the superiority of the physical dose distribution of IMRT compared to 3DCRT in the treatment of gastric cancers [21, 22].

The comparison of 3F-CRT and 4F-CRT plans showed that the dose coverage of PTV in both cases was similar and comparable. But as expected from the use of the additional PA field in 4F-CRT, the dose to liver was less compared to the 3F-CRT plan, while the doses to the kidneys and spinal cord were slightly more in 4F-CRT plans than in 3F-CRT plans. However, this marginal dose differences in OARs would not give overall clinical significance between the two techniques. Depending upon the shape and the size of the PTV and critical organs, by comparing the DVH values, either one of the plans can be chosen.

In this study, the PTV from IMRT plans showed a systematic and significant improvement in terms of target coverage and homogeneity compared to 3D-CRT plans. Conformity index is used to evaluate the clinical evidence of better treatments. The results showed that IMRT treatment plans give considerable improvement of dose conformity to PTV with higher value of CI compared to both 3F-CRT and 4F-CRT plans. Improved conformity may help to deliver higher doses to the PTV without delivering more doses to the surrounding normal tissue. This was clearly demonstrated by the isodose distributions and DVH curves shown in Figures 1 and 2, respectively. The uniformity index values calculated for the target volume also showed significant advantage of IMRT plans over 3D-CRT plans.

With respect to all OARs, IMRT is able to keep the mean dose below their tolerance levels in contrast with 3D-CRT. This dose reduction in non-target structures without compromising the dose in the target volumes could lead to additional clinical advantages, because side effects during or following treatment might be reduced. On the other hand, dose reduction in the OAR volumes could allow additional dose escalation to the target structures. Therefore the capability of dose conformity of IMRT is clinically beneficial to the patients of gastric cancer.

With IMRT treatments, the mean dose to the liver was reduced by 9.6% and 5% when compared with the 3F-CRT and 4F-CRT plans respectively. This reduction of dose may appear small, but the mean values of D_2/3_, D_1/3_ and percentage of volume receiving more than the tolerance limit were reduced considerably compared to both 3D-CRT plans, which helps in reducing the toxicity.

In the case of kidneys and spinal cord, though there was a smaller difference in the mean doses with the plans of three techniques, mean values of D_2/3_,D_1/3_ and percentage of volumes receiving more than their tolerance limit were reduced significantly with IMRT (*p*<0.0001) plans. The tendency of reduction in doses to OARs with IMRT obtained in this study was similar to that of earlier studies reported by Milano et al. [23] and Kataria et al. [24].

The bowel contour consisting of small and large intestines was done and no constraints were given in IMRT plans. Since it is a mobile organ, the authors wanted to verify the dose distribution and compare the values with these three techniques. The results showed that all the dose parameters of bowel in three field and four field conformal plans were comparable and IMRT plans reduced them considerably. The reduction of dose in OARs with IMRT plans may be due to the use of more number of fields with appropriate angle selection, which causes reduction of entrance and exit dose to those organs.

## CONCLUSION

IMRT plans improve the homogeneity and conformity of dose distribution in the target volume. The uniformity index and standard deviation (SD) values also confirmed a better 3D dose homogeneity in the PTV with the IMRT technique. With this method, the maximum dose coverage around the PTV has reduced considerably. In the treatment of gastric malignancies IMRT reduces the mean dose and the dose above threshold to critical normal tissues, particularly to the liver and kidneys. With this technique, the desired dose distribution can be achieved due to its ability to provide sharp dose gradients at the junction of target volume and the adjacent OARs. Overall, the doses to all the OARs were lower for IMRT plans and were in acceptable limits. The advantages of IMRT plans include both improved planning target volume coverage and improved sparing of critical organs.

In conclusion, this study suggests a dosimetric benefit of IMRT over conventional 3D-CRT planning and indicates the importance of IMRT in the adjuvant treatment of gastric cancer. The study helped the authors to understand the role of IMRT in detail and created confidence to consider the same to treat the patients with carcinoma of gastric cancers. However, the superiority of IMRT over conventional 3D-CRT must be mitigated with the caution for organ motion. Currently the authors are studying the effect of organ motion in the upper abdomen as a prerequisite for the use of IMRT for patient treatment to further evaluate doses received by these moving organs.
